# Correlation of Breakthrough Infection During the Omicron Wave With Seropositivity of Vaccinated Patients Undergoing Hemodialysis

**DOI:** 10.7759/cureus.29296

**Published:** 2022-09-18

**Authors:** Aswini P Patnaik, Nikunj K Rout, Sakir Ahmed, Kumar A Dash, Ashok K Praharaj, A. Raj K Patro

**Affiliations:** 1 Nephrology, Kalinga Institute of Medical Sciences, Bhubaneswar, IND; 2 Rheumatology, Kalinga Institute of Medical Sciences, Bhubaneswar, IND; 3 Microbiology, Kalinga Institute of Medical Sciences, Bhubaneswar, IND; 4 Molecular Biology, Kalinga Institute of Medical Sciences, Bhubaneswar, IND

**Keywords:** enzyme linked immunosorbent assay (elisa), rt-pcr, antibody, covid-19, dialysis, breakthrough infection, sars-cov-2

## Abstract

Background

Patients with chronic kidney disease and undergoing hemodialysis are at greater risk of developing COVID-19. In spite of vaccine efficacy, SARS-CoV-2 breakthrough infection has been reported in several studies. This study was carried out to assess if seroconversion could predict SARS-CoV-2 breakthrough infection in a cohort of vaccinated patients undergoing hemodialysis.

Methodology

Patients undergoing maintenance hemodialysis for at least three months and who had received two doses of BBV152 or AZD1222 vaccine were included in the study. Their baseline IgG antibodies to SARS-CoV-2 were measured and followed up for a median of three months during the third wave of COVID-19 in India with SARS-CoV-2 reverse transcription polymerase chain reaction (RT-PCR) to detect breakthrough infections.

Results

Of 80 patients enrolled, seroconversion was seen in 81% of the cases, and SARS-CoV-2 breakthrough cases have been detected in 16% (13/80; 95% CI 8.95-26.18) patients undergoing hemodialysis. Of the 13 patients, seven patients required hospitalization and others had a mild outcome. There was no correlation of baseline seropositivity with breakthrough infections or hospitalization.

Conclusions

A majority of patients who underwent hemodialysis are seropositive post-vaccination. The breakthrough infection did not correlate with baseline seroconversion. Thus, there would be other predictors of breakthrough COVID-19 infections that need to be recognized in this susceptible population.

## Introduction

Chronic kidney disease (CKD) is an independent factor leading to severe coronavirus disease (COVID-19). CKD patients may also have hypertension and diabetes, which are also predictors of worse outcomes in COVID-19 [1-3]. A meta-analysis has shown that patients on hemodialysis (HD) are at higher risk for COVID-19 [4]. Furthermore, the levels of neutralizing antibodies were significantly lower in hemodialysis patients compared to the general population and can be an additional risk factor. As of August 26, 2022, there have been 596,873,121 confirmed SARS-CoV-2 cases globally, and 6,459,684 deaths have been reported [5]. In India alone, 44,389,176 cases have been reported, with 527,556 deaths [6]. The vaccination program for SARS-CoV-2 in India started in January 2021. During this time period, two vaccines, Covaxin (BBV-152, inactivated vaccine made by Bharat-Biotech, Hyderabad, India) and Covishield (AZD1222, recombinant vaccine made by Serum Institute of India, Pune, India), were approved by the Indian Council of Medical Research for vaccination [7-8]. The indigenous developed Covaxin vaccine (BBV152) is a SAS-CoV-2 whole virion inactivated vaccine adjuvanted with an imidazoquinoline molecule chemisorbed on alum (Algel-IMDG). The Algel-IMDG intended to direct vaccine antigen directly to draining lymph nodes. The Covishield vaccine is a replication-deficient chimpanzee adenovirus vector, which encodes the Spike (S) glycoprotein of SARS-CoV-2. The Covaxin vaccine is tolerable in safety outcomes and has been shown to have enhanced immune responses [7-10]. In spite of vaccine efficacy, SARS-CoV-2 breakthrough infection has been reported in several studies in healthcare workers and otherwise healthy individuals [11-12]. However, Information on the antibody response to SARS-CoV-2 and breakthrough infection in hemodialysis patients is limited.

We wanted to know if the pre-existing antibodies in vaccinated patients on hemodialysis could protect against breakthrough infections. This study was carried out to measure the SARS-CoV-2 breakthrough infection by RT-PCR in a cohort of vaccinated patients undergoing hemodialysis.

## Materials and methods

This was a cohort study of patients undergoing hemodialysis. A baseline seroprevalence was estimated using sera of patients who had completed two doses of either COVID-19 vaccine (BBV152 or AZD1222) before the onset of the third wave of COVID-19 in India, presenting to the nephrology department of Kalinga Institute of Medical Sciences, a tertiary care hospital in Bhubaneswar, India. The time interval between two doses of vaccine was four to six weeks for BBV152 and 12 to 16 weeks for AZD1222. A convenient sample size was included in the study (n= 80). These patients were followed up for the next three months to find out how many developed breakthrough infections. Any patient that had any infection requiring antibiotics, received any immunosuppressant drugs, or was hospitalized in the last three months was excluded. This study was approved by the Institute Ethics Committee of Kalinga Institute of Medical Sciences (KIMS), and samples were collected after obtaining informed written consent. Relevant demographic and clinical information was obtained from patients and documented. Blood samples were collected to detect IgG antibodies to SARS-CoV-2 in sera and were measured by an enzyme-linked immunosorbent assay (ELISA, five to six months after the second dose) in December 2021 before the onset of the third wave in India. Every week, when the patients came for an HD, nasopharyngeal swabs were taken and processed for the detection of SARS-CoV-2 by reverse transcription polymerase chain reaction (RT-PCR).

IgG antibodies to SARS-CoV-2 in sera were measured by an enzyme-linked immunosorbent assay (ELISA) using the Covid Kawach IgG Microlisa kit (J. Mitra & Co. Pvt. Ltd. New Delhi, India; kit approved by the Indian Council of Medical Research) as per manufacturer’s instructions [12, 13]. In brief, the IgG antibodies present in the serum samples bind with the SARS-CoV-2 antigen coated in the ELISA plate, followed by the addition of anti-human IgG (horseradish peroxidase-labeled) to capture human IgG antibodies. Then a chromogenic substrate 3,3’,5,5’ -tetramethylbenzidine (TMB) was added, and the reaction was stopped by the addition of 1N H2SO4. The plate was then read at 450 nm [13, 14].

Molecular detection of SARS-CoV-2 RNA by RT-PCR was carried out using the Taqman probe-based method as described earlier [15]. In brief, a nasopharyngeal sample (NPS) was collected for the detection of SARS-CoV-2 by RT-PCR. The NPS specimens were collected by a healthcare professional and the swabs were placed in a sterile transport tube containing 2 ml of virus transport medium and were transferred to a laboratory on an ice pack at 4C. RNA was extracted from swab samples using a bead-based RNA extraction system in the KingFisher Duo system (Thermo Fisher Scientific, Waltham, Massachusetts). The extracted RNA was subjected to real-time reverse transcription polymerase chain reaction (RT-PCR) using the CoviPath COVID-19 RT-PCR kit (Thermo Fisher Scientific, Waltham, Massachusetts) as per the manufacturer’s instructions. Each reaction included a SARS-CoV-2-specific positive control, negative control, and extracted RNA template in a 25 µl reaction volume. This kit detects nucleocapsid (N) gene and open reading frame 1 (ORF-1) target of SARS-CoV-2. The ribonuclease (RNase) P target was used as an internal control in the reaction. A Ct value of <37 for the target gene was considered positive for SARS-CoV-2 [15]. SARS-CoV-2 RNA detection by RT-PCR performed on >11 or more days after completion of both doses of vaccination was considered a breakthrough infection [16, 17].

Statistical analyses were performed using software GraphPad Prism®software PRISM version 6 (GraphPad Software, San Diego, California). A p-value of <0.05 was considered as significant.

## Results

**Figure 1:**
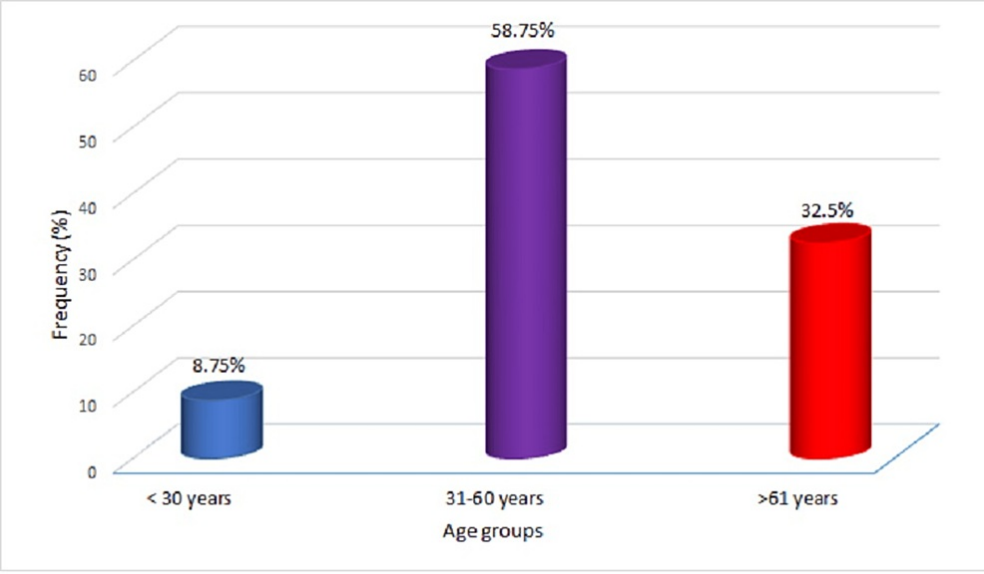
Age-specific prevalence of IgG antibodies to SARS-CoV-2 in hemodialysis patients (n=80)

Eighty hemodialysis patients’ serum samples were assayed for SARS-CoV-2 IgG detection by ELISA. These patients were followed up for the next three months and NPS samples were obtained every week and tested for detection of SARS-CoV-2 by RT-PCR. The patients included 61 (76.25%) males with a mean age of 46±13.57 years. Patients’ age range was from 21 to 72 years; most patients were in the age group of 31 to 60 years. Age-specific prevalence is depicted in Figure 1. All patients had presented with end-stage renal disease (ESRD) and hypertension (HTN), and 34% of patients had diabetes mellitus. One patient had undergone a coronary artery bypass graft (CABG). The patients were on dialysis twice weekly. The demographic details are provided in Table 1. A majority of patients received the indigenously developed Covaxin vaccine. Out of 80 subjects enrolled in this study, 76 administered Covaxin, whereas only four subjects received Covishield. The overall seroprevalence of SARS-CoV-2 was found to be 81% (95% CI 70.97-8 9.11) in the patients on HD. During the third wave in India (January-March 2022), 13 patients turned out to be positive for SARS-CoV-2 by RT-PCR, and seven patients underwent hospitalization due to of COVID-19. Thus, SARS-CoV-2 breakthrough cases were documented in 16% (13/80; 95% CI 8.95-26.18) (Table 2). Among these seven patients hospitalized due to COVID-19, two seronegative patients became positive for SARS-CoV-2. There was discordance between baseline seropositivity and breakthrough infections (Table 2). The seropositivity was not associated with severe COVID-19 as measured by the need for hospitalization (Table 3). Out of these, three patients died during the study period due to COVID-19-related illness. The other patients had mild symptoms or were asymptomatic.

**Table 1:** Demographic distribution of patients undergoing hemodialysis (n=80) HTN - hypertension, T2DM - diabetes mellitus; data presented as mean ± SD or n (%)

Demographic profile and parameters	Hemodialysis patients
Age in years (mean)	46 ± 13.57
Male sex	61 (76.25%)
Female sex	19 (23.5%)
Weight, kg	63.3 ± 5.5
Hemoglobin, g/dL	8.4 + 1.06
Serum albumin, g/dL	3.26 ± 0.3
Urea, mg/dL (mean)	99.85 ± 14.56
Serum creatinine, mg/dL (mean)	8.33 ± 1.24
Frequency of dialysis (weekly)	2
HTN	80 (100%)
T2DM	27 (33.75%)
SARS-CoV-2 vaccination (BBV-152 / AZD1222	80 (100%)

**Table 2:** SARS-CoV-2 breakthrough infections versus seropositivity (n=80)

	Not infected	Breakthrough infection	p-value
Seropositive	54	11	0.734
Seronegative	13	2	

**Table 3:** SARS-CoV-2 breakthrough infections and need for hospitalization versus seropositivity (n=13)

	Not hospitalized	Hospitalized	p-value
Seropositive	6	5	0.224
Seronegative	2	0	

## Discussion

This study aimed to assess if SARS-CoV-2 breakthrough infection could be predicted by a previous seropositivity. Patients with co-morbid conditions, like ESRD, and who are undergoing hemodialysis are at greater risk of developing COVID-19. Although SARS-CoV-2 vaccines are effective in the prevention of the majority of cases, due to emerging variants, it is not effective in 100% of cases, and breakthrough infections have been reported. Vaccine breakthrough infections have been reported in several different populations; however, data on the hemodialysis select group is limited. Bergwerk et al. [16] reported COVID-19 breakthrough infection in 2.06% of healthcare workers. A study from India showed that the prevalence of breakthrough infections was 16.9% after the completion of both doses of vaccination [17]. Another analysis of breakthrough infections from India had shown reducing protective antibody titers, and there was a correlation of protection between antibody titers and breakthrough infections [18]. However, this study had been carried out prior to the Omicron wave and had a lower incidence of breakthrough infections (7.4%). In patients on dialysis in the United Kingdom, breakthrough infections were 17.8%. This was carried in nearly the same time as our study, and this can explain the similar incidence of breakthrough infections. The majority of this UK cohort also had had two doses of vaccinations, and only those with three doses had shown a better rate of protection [19, 20]. Previous studies have shown that antibody titers can predict breakthrough infections [18]. There is some data on breakthrough infections in hemodialysis patients from before the Omicron wave. One study before the Omicron wave reported breakthrough infections in 6% of patients on maintenance HD and 4.3% of kidney transplant recipients [21]. A much large study reported this to be around 2% [19]. This study by Anand et al. showed that antibody titers wane rapidly in patients on HD. And all of these studies looked at variants before Omicron. There is some evidence that heat-killed vaccines may provide lesser protection in immunocompromised patients. Also, the omicron wave had higher breakthroughs, especially in patients who had received only two doses of vaccines [11,18,19]. In India, unlike in some other countries, booster shots were not recommended until after the Omicron wave.

The Omicron variant is notorious for evasion of previous anti-SARS-CoV-2 antibodies [11]. Other studies have also reported that post-vaccination antibodies may not neutralize Omicron [12]. It has also been observed that a longer gap in vaccination and infection could provide better protection against the Omicron variant [22]. Thus there is a unique challenge to the Omicron variant in finding a biomarker to predict breakthrough infections. This leads to the question of whether this phenomenon is unique for the Omicron variant or whether other potentially emerging variants may also have such escape from antibodies. The other question remaining is whether there is some other biomarker than can assess the effectiveness of COVID-19 vaccines. Future studies are needed to answer these questions.

This study has limitations. In this study, we have measured and used the qualitative detection of IgG antibodies to SARS-CoV-2 and measured the breakthrough infection by molecular detection by RT-PCR. We have not used the quantitative measurement of IgG against SARS-CoV-2. Other limitations of this study are the small sample size, and since the IgG measured is not quantitative, without knowing peak antibody response, we cannot assume that seroconversion alone is enough to confer protection against COVID-19. In addition, we have not sequenced the breakthrough sample to confirm the Omicron variant. However, at the time of the study (January-March 2022), Omicron was the dominant strain in India, accounting for more than 90% of COVID-19 infections [23]. This study identified that a majority of patients undergoing dialysis have IgG antibodies to SARS-CoV-2. This has various implications. First, breakthrough infections occur in HD patients even after full vaccination. Second, seroconversion does not predict protection from breakthrough infection. Third, it raises questions about whether emerging variants will lead to more breakthrough infections. One reason for our findings may be due to the rapid waning of the antibody response. This leads to the question of the optimum timing of boosters and if they need to be repeated [24].

Information on seroprevalence data will be useful in patient stratification to avoid virus transmission and in the management of patients. Further, more studies from different geographic regions are warranted to get the true picture of the seroprevalence/breakthrough infection of SARS-CoV-2, which will guide for devising strategies for a booster dose in this select population.

## Conclusions

The main result of this study is that the post-vaccination seroconversion status could not predict susceptibility to breakthrough infections. This is important for clinicians managing patients with chronic kidney diseases because they need to find other correlates of protection in this group. Also, further strategies may be needed, such as additional booster doses. A majority of patients who underwent hemodialysis have antibodies against SARS-CoV-2. Sixteen percent of hemodialysis patients reported having a breakthrough infection. This underscores the need to optimize the timing for a booster dose in patients undergoing dialysis. Also, there is a need for a practical biomarker to assess vaccine effectiveness since antibody levels are not useful in this special population.
